# Immunogenetic Mechanisms Leading to Thyroid Autoimmunity: Recent Advances in Identifying Susceptibility Genes and Regions

**DOI:** 10.2174/138920211798120790

**Published:** 2011-12

**Authors:** Oliver J Brand, Stephen C.L Gough

**Affiliations:** Oxford Centre for Diabetes Endocrinology and Metabolism (OCDEM), Oxford, UK

**Keywords:** Autoimmune thyroid disease, Graves' disease, association, genes.

## Abstract

The autoimmune thyroid diseases (AITD) include Graves’ disease (GD) and Hashimoto’s thyroiditis (HT), which are characterised by a breakdown in immune tolerance to thyroid antigens. Unravelling the genetic architecture of AITD is vital to better understanding of AITD pathogenesis, required to advance therapeutic options in both disease management and prevention. The early whole-genome linkage and candidate gene association studies provided the first evidence that the HLA region and *CTLA-4* represented AITD risk loci. Recent improvements in; high throughput genotyping technologies, collection of larger disease cohorts and cataloguing of genome-scale variation have facilitated genome-wide association studies and more thorough screening of candidate gene regions. This has allowed identification of many novel AITD risk genes and more detailed association mapping. The growing number of confirmed AITD susceptibility loci, implicates a number of putative disease mechanisms most of which are tightly linked with aspects of immune system function. The unprecedented advances in genetic study will allow future studies to identify further novel disease risk genes and to identify aetiological variants within specific gene regions, which will undoubtedly lead to a better understanding of AITD patho-physiology.

## INTRODUCTION

The complete spectrum of autoimmune diseases affect the majority of tissues within the body, including, for example, pancreatic beta cells in type 1 diabetes (T1D), synovial joint antigens in Rheumatoid Arthritis (RA) and myelin surrounding nerve axons in Multiple sclerosis (MS) [1, 2]. Although the epidemiology varies according to individual conditions, collectively, autoimmune prevalence is at least 5% in the general population and is one of the major causes of premature mortality in young and middle aged women [2, 3]. By far the most common are the autoimmune thyroid diseases (AITDs) [2, 4], which include Graves’ disease (GD) and Hashimoto’s thyroiditis (HT), both characterised by lymphocytic infiltration of the thyroid with autoantibodies targeting thyroid antigens, including thyroid peroxidise (predominantly in HT), thyroglobulin (Tg) and the thyroid stimulating hormone receptor (TSHR) [5, 6]. Despite a similar autoantibody profile GD and HT display distinct clinical phenotypes. Lymphocytic infiltration in HT ultimately leads to thyroid cell destruction and hypothyroidism [5]. In contrast the net effect in GD is usually autoantibody stimulation of the TSHR, independent of its ligand thyroid stimulating hormone (TSH), resulting in hyperthyroidism [6]. Both diseases are typical multifactorial disorders with a complex aetiology involving both genetic and environmental factors, with twin studies suggesting that up to 80% of risk may be attributed to genetic factors [7, 8].

**Fig. (1):**
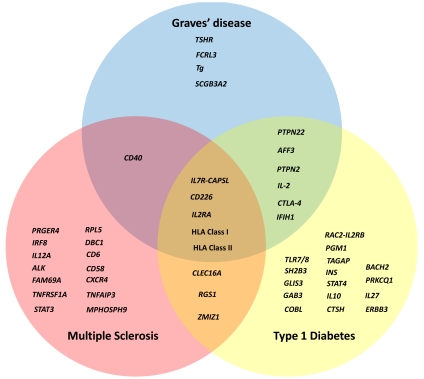
**Sharing of susceptibility genes among the Autoimmune diseases, Graves’ disease, Multiple sclerosis and Type 1 diabetes.** The figure shows disease risk genes that are unique to the 3 individual diseases and those shared between either two of the diseases or all three disorders.

The well documented co-clustering of autoimmune diseases within families and individuals, together with apparent sharing of a number of disease risk genes (Fig. **1**) [9-2] suggests at least some common disease mechanisms. Improving our understanding of AITD pathophysiology could lead to better therapeutic options not only for disease management and/or predictive models for AITD but could also have implications for other autoimmune disorders. Genetic studies over the last 20 years have aimed to identify loci that confer susceptibility to AITD as a strategy to better understand the underlying biology behind disease. During this time numerous advances have been made in our understanding of AITD genetics, particularly in GD. This review will provide a brief background of AITD genetics, followed by a more detailed examination of AITD risk genes that have recently been more extensively mapped for association with GD. This will be followed by an assessment of more recent disease gene discovery efforts, with a final broad overview of pathways involved in GD pathogenesis uncovered through genetic studies.

## EARLY DAYS OF AITD GENE DISCOVERY

Initial efforts to identify disease risk genes employed both, family-based linkage studies, examining the co-inheritance of microsatellite markers (1-6bp tandem repeat sequences) between affected and non-affected family members, and candidate gene association studies in which the frequency of di-allelic single nucleotide polymorphisms (SNPs) were analysed in unrelated disease affected cases and unaffected controls. Both approaches were initially hampered because of inadequate knowledge of human genetic variation, undersized study cohorts and a lack of high throughput genotyping methodologies with a general reliance upon PCR amplification followed by restriction enzyme digestion. A major step forward was the first detailed linkage map of microsatellite markers across the entire human genome heralding a new era of large-scale, genome-wide linkage studies, which it was hoped would yield novel disease susceptibility genes in complex disease [13].

**Fig. (2):**
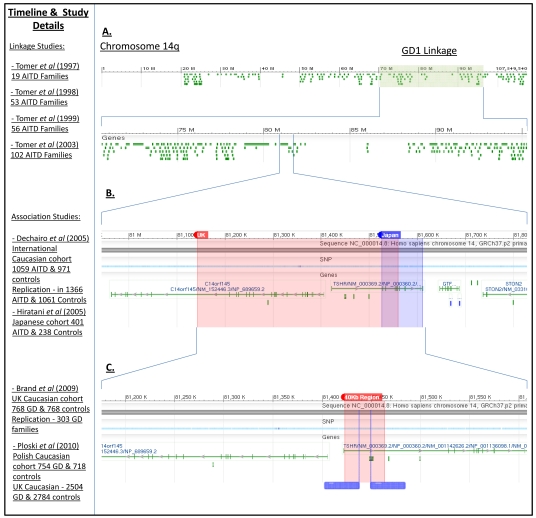
**Timeline of narrowing TSHR association with Graves’ disease**. **A**. the GD-1 region of linkage spanning more than 25Mb of chromosome 14q. **B**. The shaded regions represent the boundaries of all SNP associations identified by Dechairo *et al*. [56] in a case-control cohort of international Caucasian decent and the boundaries of association identified by Hiratani *et al*. (2005) [57] in a Japanese cohort. **C**. The highly associated 40Kb region within *TSHR* intron 1 with the two most strongly associated SNPs marked within the region (rs179247 and rs12101255). Images were generated using the NCBI sequence viewer (http://www.ncbi.nlm.nih.gov).

The first linkage study performed in a complex genetic disorder was in T1D [14, 15], shortly followed by linkage analysis in most multifactorial diseases, including AITD [16-20]. Despite the successful use of linkage studies to identify rare and highly penetrant mutations in monogenic disorders, such as cystic fibrosis [21] and maturity-onset diabetes of the young [22, 23] this approach largely failed to deliver novel susceptibility loci in AITD. The first linkage study in AITD consisted of 56 multiplex Caucasian family members (families with either GD or HT as well as unaffected members) and identified linkage at 7 regions, 3 were GD specific termed, GD-1 (14q31) (GD-1 locus displayed in Fig. **2A**), GD-2 (20q11.2), GD-3 (Xp21), 2 in HT called HT-1 (13q32) and HT-2 (12q22) and one common AITD locus, AITD-1 (6p11) [16]. Further investigation using the same cohort with 46 additional families (102 total) failed to replicate linkage at three of these loci, Xp21, 13q32, 20q11.2 and signals detected at 6p11 and 14q31 were detected many centimorgans (cM) distant from the original markers, raising concerns over reproducibility of results [17, 24]. The largest AITD genome linkage screen to date investigated 389 microsatellite markers in 1119 Caucasian AITD relative pairs from the UK, continental Europe and Australasia revealing suggestive evidence of linkage with multi-point logarithm of odds (LOD) scores between 2 and 3 at chromosomes 18p11, 2q36 and 11p15 [18], but failed to replicate the previous loci [16]. Chromosome 5q31-q33 identified by independent linkage studies in Japanese and Chinese cohorts represented the most convincing evidence that an AITD predisposing gene might be located within the region [19, 20]. A study in 123 Japanese sib-pairs investigated 392 microsatelite markers across the genome and identified the strongest multipoint LOD score of 3.14 at D5S436 within 5q31-q33 [19]. This was subsequently replicated in 54 Han Chinese multiplex families producing a LOD score of 2.8 for D5S436 [20]. Investigation of four additional markers surrounding D5S436 identified strongest linkage at nearby D5S2090 giving a 2-point LOD score of 4.31 [20]. With the exception of 5q31-q33 the majority of signals detected in all studies fell below linkage LOD scores acceptable for genome-wide significance (LOD>3) highlighting the uncertainty in results produced [15]. Furthermore, the low coverage of linkage maps meant the physical genomic distance between markers was large making it very difficult to determine the causal gene in a region of linkage. In retrospect failure to identify now well recognised susceptibility genes or regions such as HLA, *CTLA-4* and *PTPN22* reflects the disappointing application of this approach. These studies suggest that in AITD, like most other complex diseases, there are unlikely to be any or at least very few causal or highly penetrant common variants that directly induce AITD in certain individuals.

Association studies inherently have greater power to detect a disease-risk gene, since greater numbers of SNPs can be investigated in a single region and large, well-powered case-control cohorts are more easily collected. The limitation however, is that a causal variant must be either directly interrogated or a neighbouring marker that is strongly correlated with the aetiological variant be tested to detect association. Before the arrival of improvements that would later facilitate genome-wide association studies (GWAS), researchers investigated candidate gene regions, often focusing on small numbers of variants where functional effects could be more easily translated, such as non-synonymous (amino acid altering) SNPs (nsSNPs) or those located in 3’ or 5’ regulatory regions [25-27]. Accumulation of data from multiple studies gradually provided convincing evidence for two AITD susceptibility loci; HLA and *CTLA-4* [27-32]. For more than 30 years evidence suggested the HLA region on 6p21 predisposed to autoimmune diseases [33-35], although early studies provided inconclusive evidence of association with GD [36-41]. However, studies eventually provided good evidence that alleles within the HLA class II region were strongly associated with GD [28, 29]. Further investigations have since refined the major association signals to genes within the HLA class I region, *HLA-C* and *HLA-B* [42] and the HLA class II region genes *DRB1* and *DQA1 *[43, 44]. Evidence of *CTLA-4* association with GD was demonstrated by a number of studies investigating common functional variants, including a dinucleotide (AT)n repeat within the 3’ UTR [27], a single SNP in the promoter region 318bp upstream of the start codon [31], an exon 1 SNP [30] and a non-coding intron 1 variant [32]. Comprehensive fine-mapping of the *CD28-CTLA-4-ICOS* region has since refined the association signal to within a 6.1Kb region, 3’ of *CTLA-4*, with 4 SNPs being strong aetiological candidates [9]. Despite these two successes, investigating a limited number of variants in a given region led to some genes being incorrectly ruled non-associated with AITD, such as the *TSHR*, which was later confirmed as being associated in more recent detailed investigations. 

## LATER DAYS OF AITD GENE DISCOVERY AND REFINING ASSOCIATION SIGNALS

Completion of the first human genome sequence just more than a decade ago provided a reference to begin cataloguing the true extent of genetic variation, much needed for improved disease gene discovery. The International HapMap Project represented the first major undertaking, which characterised 3.5 million common SNP allele frequencies and the correlation between them or linkage disequilibrium (LD) in several ethnic populations (www.hapmap.org). LD can be defined as the naturally occurring, non-random clustering of alleles at neighbouring SNPs. LD mapping across the entire human genome has enabled researchers to carefully select a series of tag SNPs that capture the genetic variation of correlated un-typed SNPs and thus remove the need to genotype every SNP in a given region [45-49]. Further developments in genotyping technology, moving from PCR-RFLP based methods to faster and more cost effective fluorescent probes has enabled larger numbers of SNPs to be interrogated in a single study. In addition, collection of larger AITD cohorts by several investigators provided greater power to detect regions of association, which was important, considering the modest magnitudes of effect anticipated (OR=1.2-1.5) [50-52]. These advances have facilitated comprehensive SNP screening and enabled both novel disease gene identification and refining of association signals by interrogating a dense set of common SNPs to narrow down the location of the aetiological variant. Notable examples include the* TSHR*,* SCGB3A2,* *PTPN22*, and *IL2Rα*. 

### TSHR

The *TSHR* encodes the primary autoantigen in GD and as such represents a prime candidate gene. Interestingly, a number of microsatellite markers that define a 25cM region of linkage to GD, termed GD-1, encompasses the *TSHR* as well as a number of other plausible candidate genes (Fig. **2**) [16, 17, 24, 53, 54]. Several early association studies on *TSHR* nsSNPs were performed, however these provided conflicting evidence of *TSHR* association with GD [55]. More convincing evidence of *TSHR* association with GD, but not HT, came from independent tag SNP screens in Japanese and Caucasian cohorts, which identified two overlapping signals [56, 57]. Twenty two *TSHR* intronic SNPs investigated in 401 AITD patients (250 GD and 151 HT) and 238 controls of Japanese descent identified several individual SNP and haplotype associations across the region [57]. Strongest SNP associations were located within intron 7 of the *TSHR* (P=10^-2^-10^-4^) [57]. In contrast, 20 SNPs spanning an extended 600Kb of the *TSHR* were analysed in a multinational Caucasian cohort, which identified 10 SNP associations located across a wide 340Kb region encompassing* TSHR* intron 1 and the neighbouring 5’ *C14ORF145*, approximately 140Kb upstream of the main signal identified in Japanese [56]. More recently a panel of 98 SNPs selected across 800Kb encompassing *C14ORF145-TSHR-STN2-GTF2A1* were investigated in 768 UK Caucasian GD cases and controls [58] (Fig. **2**). Numerous SNP associations were identified, with SNPs rs179247 and rs12101255 demonstrating strongest evidence of association with GD, and which are located within a distinct 40Kb of *TSHR* intron 1 [58]. These variants have since been further replicated in independent UK and Polish case-control cohorts (P=10^-15^ – 10^-21^ and odds ratios (OR) = 1.38 – 1.87), with logistic regression suggesting that the aetiological variant is most likely to be in strong LD with rs12101255 [50] (Fig. **2**). All variants within and just outside the 40Kb region of *TSHR* intron 1 now require detailed investigation to identify the aetiological variant within the region.

### SCGB3A2

Replicated in two independent linkage studies, the previously discussed chromosome 5q31-q33 region demonstrated the most convincing evidence of linkage in AITD [19, 20]. Across this region there are a number plausible AITD candidate genes, including; interleukin 12b (*IL-12b*) [59, 60], *IL-13*, *IL-4* [61-63], interferon regulatory factor 1 (*IRF1*) and beta-2-adrenergic receptor (*ADRB2*) [64], exemplifying the challenge involved in identifying a causal gene in a region of linkage. To locate the causal gene, Song and colleagues recently performed a comprehensive series of association screens to identify the Secretoglobin Family 3A Member 2 (*SCGB3A2*) gene [51]. An initial screen of 179 SNPs encompassing 3Mb around the AITD linked marker, D5s2090, in 384 GD cases and controls from Shandong, China, identified strongest SNP association (rs1368408) within the promoter of* SCGB3A2* [51]. To refine association, a further 122 SNPs were identified by re-sequencing and investigated in two independent Chinese case-control cohorts [51]. Strongest SNP association remained within the *SCGB3A2* promoter SNP, rs1368408 and a second neighbouring SNP (P=10^-5^-10^-6^, OR=1.28-1.32) in the combined Chinese cohort (2811 GD patients and 2807 controls) [51]. Importantly the most highly associated SNP (rs1368408) has since been replicated in two independent Caucasian GD cohorts; the first from the UK National collection of AITD cases, consisting 2504 GD and 2688 control from the 1958 British Birth Cohort (58c) (P=10^-3^, OR=1.18) and the second in 1474 GD and 1619 controls from Russia (P=10^-5^, OR=1.33), confirming that SCGB3A2 confers predisposition to GD [52, 65]. Although there is little doubt *SCGB3A2* is a genuine GD susceptibility locus, it remains possible other genes within the region may also confer an independent risk to GD. A recent study performed a tag SNP screen of 19 candidate genes within 5q31-q33 in 428 GD cases and 690 controls of South Han Chinese descent [66]. Although *SCGB3A2* was not one of the 19 genes selected for analysis the study identified association with an *IL-3* nsSNP (rs40401), which was subsequently replicated in a second independent Han Chinese cohort (P=10^-3^-10^-4^, OR=1.21-1.33), suggesting *IL-3* might be a second susceptibility gene within the region [66].

### PTPN22

Accumulating evidence of shared susceptibility loci among the autoimmune diseases has directed a number of other gene discovery efforts in AITD, including *PTPN22,* now a well-established general autoimmune risk locus. *PTPN22* located on chromosome 1p13 encodes the cell signalling protein, lymphoid tyrosine phosphatase (LYP) which is a potent regulator of T cell activation [67]. The nsSNP located at 1858bp (C/T) within *PTPN22* results in substitution of Arginine (Arg) (CGG) to a tryptophan (Trp) (TGG) at codon 620 (R620W) (rs2476601) and was first associated with T1D susceptibility in two independent case-control cohorts of North American (P=10^-3^) and Sardinian (P<0.05) background [68]. A follow up study confirmed *PTPN22* R620W association in case-control and family T1D cohorts and provided the first evidence that *PTPN22* was a general autoimmune risk locus by demonstrating strong association in 901 UK Caucasian GD and 833 ethnically matched controls (P=10^-4^, OR=1.43) [69]. At the same time the 1858T allele was associated in an independent GD cohort (P=3.4x10^-5^, OR=1.88) [70]. Further studies have replicated association with GD and have also identified association in HT patients [12] as well as in other autoimmune diseases, including RA and Systemic Lupus Erythematosus (SLE) [71, 72]. While tag SNP screening in GD suggested effects independent of R620W [73], based on size of association compared to surrounding variants and functional studies performed it remains likely that R620W is the aetiological variant. 

### IL2Rα

The interleukin-2 receptor alpha (IL2Rα)/CD25 gene was first investigated in T1D [11] and followed up in GD [10]. *IL2Rα* encodes one of three proteins (the other 2 being IL-2Rβ and IL-2Rγ) that make up the interleukin-2 receptor (IL-2R) important in lymphocyte function, particularly T regulatory cells (CD4^+^ CD25^+^ FOXP3^+ ^T cells) [74]. The first evidence that *IL2Rα* confers susceptibility to T1D was demonstrated using a set of 20 tag SNPs across the gene region in large T1D case-control and family collections [11]. Using a multilocus test of association there was significant evidence for a T1D aetiological variant within the gene region (case-control P=6.5x10^-8^) [11]. The multilocus test treats the SNPs collectively and so a measure of association is generated for the entire region, which can generate greater power to detect an association compared to investigating individual SNPs in a region [75, 76]. Based on these findings our group investigated the same set of tag SNPs across *IL2Rα *in 1896 GD cases from the UK national collection and 1822 58c controls, identifying evidence of GD risk within the region (multilocus P=4.5x10^-4^) [10]. Investigations in a Japanese GD cohort recently replicated *IL2Rα* association in a single SNP (P=0.03) within the region [77]. Further studies are required to replicate association and to identify the aetiological variant in GD. Detailed fine-mapping studies in T1D have since refined the association map to 2 independent groups of SNPs showing strong association with T1D (maximal P=10^-28^, OR=2.04), the first within *IL2Rα *intron 1 and the second located 5’ of *IL2Rα* upstream of neighbouring *RBM17 *[78]. The T1D associated alleles also demonstrated strong association with reduced soluble IL-2RΑ protein [78]. Variants in *IL2Rα* are also predisposing in MS, albeit with different variants within intron 1 showing strongest association with disease [79, 80]. A recent comparison of T1D and MS association at *IL2Rα* demonstrated substantial discordance; with a T1D risk allele within intron 1 (rs41295061) not associated with MS, a T1D risk allele located 5’ of *IL2Rα* (rs11594656) conferring protection from MS and a variant conferring risk to both diseases (rs2104286) [81]. Interestingly, all alleles whether conferring, predisposition or protection to T1D or MS, were associated with reduced soluble *IL-2RΑ* protein in both patient sub-groups [81]. The paradox in which alleles confer protection and predisposition, yet all influence soluble *IL-2RΑ* protein in the same way is unclear without having a better understanding of the biological pathways involved. Similar heterogeneity between autoimmune diseases has also been observed in *PTPN22*, where the 1858T allele is associated with predisposition in T1D, GD, HT and RA, but confers protection in Crohn’s disease (CD) [82].

A number of other loci have been identified through candidate gene approaches that are likely to be true GD, HT or combined AITD risk genes, including thyroglobulin (Tg) [83-86] and CD40 [87-89], however further studies are needed to refine the association signal in these regions. Many other regions have been implicated as conferring disease risk although require independent replication to confirm association, such as *FOXP3* [90, 91] and *VDR* [92-94]. For a complete list of implicated and confirmed AITD risk loci, refer to Table **1**. Ultimately, candidate gene approaches are inherently limited by inadequate knowledge of the biological mechanisms and the function of proteins and/or non-coding RNAs encoded by the majority of genes across the genome. The arrival of genome-wide association studies (GWAS) now addresses these limitations and offers the potential to efficiently screen the genome for association in an unbiased, hypothesis free manner. 

## BRAVE NEW WORLD – GWAS AND FOLLOW-UP IN OTHER AUTOIMMUNE DISEASES

The combination of advances in our understanding of genomic diversity, collection of large disease cohorts and genotyping technology meant the potential for GWAS could finally be realised. Using detailed LD maps generated by HapMap, it was estimated that in non-African populations, approximately 500,000 (500k) SNPs across the genome would be required to screen for association and capture approximately 80% of common genetic variation [95]. Many more SNPs are required to thoroughly screen African populations since they possess much greater genetic diversity, producing much smaller blocks of LD, and therefore, a greater number of SNPs are required to capture this variation. Technological advances in 500K SNP chips made genotyping this large number of variants in large disease cohorts more cost effective. In the past few years this has led to thousands of SNPs showing reproducible association in a diverse array of complex diseases and continuous traits (http://www.genome.gov/gwastudies).

To date there has not been a full 500K GWAS performed in AITD, although results from such a study would be eagerly received. As part of the Wellcome Trust Case Control Consortium (WTCCC) of investigators, our group performed a reduced-scale association screen of 14,500 nsSNPs across the genome in 1,000 UK Caucasian GD cases, as well as the same number of breast cancer (BC), ankylosing spondylitis (AS) and MS cases with 1,500 shared 58c controls [96]. Unsurprisingly, strongest GD associations were identified within the HLA-region (P<10^-20^), which were also the largest signals in the two other autoimmune diseases, AS and MS [96]. Disappointingly no other gene regions demonstrated genome-wide significance (P<10^-5^ in a 14,500 GWAS) although modest levels of significance were identified in several non-HLA regions (P<10^-4^) [96]. Among these was a nsSNP within *FCRL3 *(P=2.1x10^-4^), which had previously been implicated as a GD susceptibility locus in Japanese and Caucasian candidate gene studies [96]. The *FCRL1-FCRL5* region on chromosome 1q23, encodes a number of FCRL genes that are known to control aspects of B cell signalling and were first identified as conferring susceptibility in RA [97]. Detailed SNP screening in RA had previously refined the association signal to four SNPs in *FCRL3*, which had subsequently been replicated in a Japanese GD cohort (n=351, P=7.4x10^-5^) with some evidence for association in HT as well (n=158, P=0.02) [97]. To determine whether the signal identified in the nsSNP screen was due to LD with variants previously identified within *FCRL3*, seven tag SNPs were selected to screen the *FCRL3-FCRL5* region in a cohort extended from 1,000 to 2,500 GD cases and 2,500 58C controls [96]. Strongest evidence of association was identified within *FCRL3* tag SNP rs11264798 (P=1.6x10^-5 ^OR=1.22) with less evidence of association in *FCRL5 *[96]. More recent association screening with logistic regression analysis across the entire *FCRL3-FCRL5* region demonstrated that the aetiological variant is most likely to be within *FCRL3* [98]. Since *FCRL3 *nsSNPs demonstrated modest significance and was later confirmed as a GD susceptibility locus, other borderline signals in this study have since been investigated. In total 10 nsSNPs demonstrated modest association (P<10^-5^) with GD and were subsequently investigated in an independent GD cohort (1578 UK Caucasian GD and 1946 controls) [99]. None of the 10 nsSNPs demonstrated GD association in the independent cohort. However after combining the data from the nsSNP study, weak association (P≥10^-3^) was identified in *HDLBP*,* TEKT1*,* JSRP1* and *UTX *[99]. Subsequent additional tag SNPs selected within these four gene regions to refine the association signal, identified additional but modest SNP associations within two of the genes, *HDLBP* and *TEKT1* (P=10^-2^-10^-3^) [99]. These 4 genes are, therefore, unlikely to confer major GD risk, however smaller risks cannot be discounted. Further investigation of these genes in independent cohorts is now required to confirm AITD susceptibility at these loci.

An advantage of the WTCCC nsSNP study was that all individuals in the 4 disease cohorts and controls were from the same ethnic background (UK Caucasian), and therefore, an additional analysis was performed, which pooled the non-AITD diseases (BC, AS and MS) and 58C together (combined controls n=4,500) to enlarge the reference control cohort [96]. This was based on the reasonable assumption that GD is likely to have at least some unique susceptibility loci from the other diseases. This analysis revealed the *TSHR* nsSNP, rs3783941, conferred strongest evidence of association with GD (P=2.1x10^-5^), confirming previous reports of *TSHR* susceptibility in GD [96]. The *TSHR* variant failed to reach even borderline significance (P=10^-3^) in the original GD versus control analysis, although greater power from an enlarged control cohort enabled identification of the well-established *TSHR* susceptibility locus [96]. This underlines the requirement for larger cohort sizes in GWAS. Despite identification of the* TSHR*, failure to identify other previously confirmed GD risk loci, such as *PTPN22,* *CD40, IL2Rα* and *SCGB3A2* reflects the lack of genome coverage in the nsSNP study. These genes may have been detected in a 500k GWAS since it is more likely that a SNP in strong LD (r^2^≥0.80) with these regions would have been directly investigated. While nsSNP screens may still have value in providing a complimentary approach, detailed association screens investigating 500,000 SNPs or more is required to sufficiently screen the human genome for GD susceptibility loci.

Recent GWAS and meta-analysis’ combined with further replication in additional cohorts has so far identified over 40 loci that affect risk to T1D [100]. The apparent sharing of susceptibility genes among autoimmune disease and the large number of risk regions now identified in T1D, prompted attempts to investigate these regions for association in GD. A follow-up study in T1D sought to investigate variants that demonstrated evidence of association, but below genome-wide significance (P<10^-7^) from 2 earlier GWAS; the first a 13,378 nsSNP screen in 3,400 T1D cases and 3,300 controls [101] and the second a 500k GWAS by the WTCCC in 2,000 T1D (as well as six other diseases) and 3,000 controls [102]. Using an independent T1D case-control (4,000 T1D cases and 5,000 controls) and second parent-child trio (n=2,997) cohort, several regions demonstrated convincing evidence of T1D association, including; C12orf30 on chromosome 12q24, *ERBB3 *on 12q13, *KIAA0350* on 16p13, *PTPN2 *on 18p11 and *CD226* on 18q22 [103]. In total 13 genomic regions showed some evidence of replication in T1D and consequently were selected for analysis in 2,200 GD cases from the UK National collection with the same controls used in the T1D study [103]. Five regions demonstrated evidence of GD association with a single SNP investigated in each locus, these were; *AFF3* on chromosome 2q11 (P=0.02, OR=1.10), *Tenr-IL2 *on 4q27 (P=0.007, OR=0.87), *CAPSL* on 5p13 (P=0.009, OR=0.88),* PTPN2* on 18p11 (P=0.02, OR=1.13) and *CD226* on 18q22 (P=0.01, OR=1.10) [103]. Further exon re-sequencing and tag SNP screening of *CD226* in T1D and MS identified a potential aetiological variant at nsSNP Gly307Ser (rs763361), which also showed strong association in 2958 GD cases and 5431 controls (P=0.03, OR=1.08) [104]. In general, the magnitudes of association for novel GD loci are quite small, and therefore, these signals require further replication in independent GD cohorts. It should be noted that in T1D much larger case-control cohorts were used to detect the association signals and as such much larger GD cohorts are likely to be required to provide more convincing evidence of association in GD. While these findings need further replication the data suggests these loci might be acting on shared autoimmune disease risk pathways.

Further work is ongoing to investigate loci identified in other autoimmune diseases for association in AITD. Determining disease pathways conferring susceptibility to autoimmunity in general, will provide novel insights into disease mechanisms and provide clues as to how specific autoimmune disorders like AITD develop. The WTCCC immunochip project, now underway, represents a major undertaking to address this, where a 200,000 custom SNP genotyping array is investigating all common variants, in regions of the genome that have previously been implicated in one or more autoimmune disease, including; T1D, GD, MS, RA, AS and SLE. This will provide a much clearer picture of susceptibility locus sharing among the autoimmune diseases, including GD and within each associated region will provide a refined association map for further genetic and functional studies.

## COPY NUMBER VARIATIONS AND FUTURE DIRECTION IN GENETIC STUDIES

The human genome contains a diverse spectrum of genetic variation from SNPs to large, microscopically visible copy number variations (CNVs), which include insertion/deletions, duplications and complex re-arrangements, ranging from >50bp to megabases (Mb) in size [105]. Since the majority of studies have focused on either SNPs or microsatellite markers, recent attention has turned to CNV analysis, to determine whether CNVs will provide novel disease association signals. A recent detailed genome screen of 3,432 CNVs were investigated in 3,000 shared controls and eight diseases, including T1D, RA, CD, type 2 diabetes, hypertension, bipolar disorder, breast cancer and coronary artery disease [106]. Just 3 susceptibility loci among the four diseases were identified; *TSPAN8* in type 2 diabetes, *IRGM* in CD and the HLA region in T1D, RA and CD, all of which had previously been identified [106]. Furthermore, it was shown that common CNVs tend to be in strong LD with SNPs present in HapMap and GWAS, suggesting previous SNP screens will have captured the majority of common genetic variation in nearby CNVs [106]. This suggests CNVs are unlikely to have utility in future screens for susceptibility loci in complex genetic diseases. However, fine-mapping studies require interrogation of all polymorphisms, including CNVs to identify an aetiological variant, and therefore, interrogation of CNVs in susceptibility regions is important [107]. The first CNV analysis in GD, recently investigated CNVs in the AITD risk regions, *CTLA-4*, *CD40* and *PTPN22* in 191 GD cases and 192 controls however no association of CNVs within these regions were identified [108]. Analysis of CNVs in other established AITD risk regions is now also required to determine whether CNVs have a role in susceptibility within these regions.

To date there are at least 10 confirmed AITD susceptibility loci with many more probable risk genes awaiting confirmation of association (Table **1**). These have predominantly been identified by SNP screens of candidate regions and follow-up studies of novel genes identified in other autoimmune diseases, such as T1D. Despite the successes, it is likely only a small proportion of genetic risk factors for AITD have so far been identified. The two major challenges in AITD genetics include; continued screening for novel AITD susceptibility loci, with the first 500k GWAS in AITD anticipated, and second, fine-mapping of associated regions to identify the key aetiological variants, which could provide clues as to how AITD risk is conferred at a given locus. The genetics field is seeing unprecedented advances; in particular next generation sequencing platforms are transforming the speed and cost of whole genome sequencing. This has facilitated ambitious ventures such as the 1000 genomes project, which aims to characterise 95% of genetic variation down to allele frequencies of 1% by sequencing well over 1000 genomes in several populations [109]. Next generation sequencing technology has also been harnessed in techniques outside the realms of genome sequencing, including; genome-scale chromatin immunoprecipitation (ChIP) followed by sequencing (ChIP-Seq) and whole exome RNA sequencing [110]. Chip-Seq data is providing detailed maps of transcription factor binding in multiple cell types and RNA-Seq will provide RNA transcript maps in many different cells and tissues, including those that are disease affected. Overlaying the many different types of data generated like Chip-Seq, onto association maps may help refine association signals and help to inform future functional studies in AITD. 

## DISEASE MECHANISMS

**Fig. (3):**
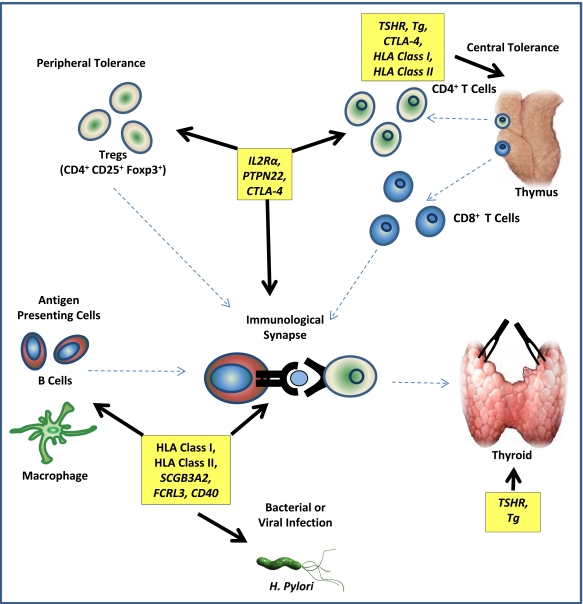
**Pathways involved in AITD pathogenesis.** The cell types and tissues believed to be involved in AITD pathogenesis. The proteins expressed by the genes or regions in boxes are implicated in various cell functions indicated by arrows. Specifically, thyroid antigens, TSHR and Tg are autoantigenic targets in GD, they are also expressed in thymus possibly for the purpose of central tolerance of maturing thymocytes and may be presented by APCs to self-reactive T cells. HLA class I and II genes are expressed in many different cell types including B cells, dendritic cells and macrophages and bind antigens for presentation to T cells during central tolerance within the thymus and in the periphery. The *IL2Rα*, *PTPN22* and *CTLA-4* are primarily expressed on various subsets of T cells whereas *SCGB3A2* and *FCRL3* are known to be predominantly involved in B cell function.

The emerging genetic architecture of AITD reflects that of other autoimmune diseases, with the HLA class I and II regions demonstrating the greatest magnitude of risk to AITD with OR>2.0 [42, 43]. The second tier of loci, with OR≥1.5, include *CTLA-4 *[9], *PTPN22* [69] and *TSHR *[50, 58], while the final group, such as *FCRL3* [96, 98] and *SCGB3A2* [51, 52, 65] exhibit more modest risk with OR=1.2-1.5. However, gene-gene interaction or epistasis and inevitably gene-environment interaction may modulate risk at individual gene regions. Improved knowledge of protein function encoded by AITD risk genes may help to answer questions of gene interaction, for example whether there are additive or multiplicative risks acquired when an individual possess a risk allele in two or more AITD predisposing genes. Although issues of interaction remain to be elucidated, a look at the AITD genes identified so far highlights a number of putative AITD disease mechanisms (Fig. **3**).

The HLA class I and II genes encode heterodimers responsible for antigen presentation in many different cell types, including B cells, dendritic cells and macrophages. The DRB1, DQA1 and DQB1 genes belong to the HLA class II region and have been consistently associated with AITD [28, 43, 44]. The predisposing DR3 haplotype, DRB1*03-DQB1*02-DQA1*0501 and protective DR7 DRB1*07-DQB1*02-DQA1*0201 have consistently demonstrated strongest association with GD [28, 29]. Further association mapping has identified amino acid substitutions at position β74 of the DRB1 binding pocket, where arginine at β74 confers predisposition and glutamine protection [43, 44]. It is possible amino acid substitutions at binding pockets like DRB1-β74 may increase the risk of antigen binding to self-antigens for presentation to CD4^+ ^T cells [111, 112]. More recently, HLA class I genes demonstrated association independently of class II regions with HLA-C and to a lesser extent HLA-B showing strongest association [42]. The HLA class I genes bind endogenously derived peptides such as bacterial and viral antigens and present to CD8^+^ T cells. This may implicate viral or bacterial infection as an environmental trigger of AITD, clearly however, further work is required to investigate this possibility. 

The strong genetic association of GD autoantigens, Tg and particularly the TSHR, suggests these proteins may not just be victims of an immune system gone wrong but may trigger or exacerbate GD in people that are autoimmune predisposed. The TSHR undergoes extensive post-translational processing resulting in an intracellular and transmembrane, B subunit, which is covalently bound to a large extracellular A subunit [113-115]. The A subunit is sometimes cleaved and shed from the membrane surface, which has been hypothesised to stimulate an autoimmune response, since the A subunit is the site of TSHR autoantibody binding, and recently characterised by X-ray crystallisation [55, 116]. Several studies have also characterised alternate TSHR mRNA transcripts, with two confirmed truncated transcripts, 1.3Kb and 1.7Kb in size, encoding nearly the entire A subunit directly, which is the target of autoantigens in GD [117-119]. These transcripts now require further functional characterisation. Expression studies in a modest number of human thyroid tissue samples (n=12) demonstrated that GD predisposing variants within *TSHR* intron 1 increase mRNA of the 1.3Kb and 1.7Kb transcripts compared to flTSHR [58]. It is therefore possible that non-coding variants may increase the level of potentially autoantigenic A subunits, which stimulate an autoimmune response in predisposed individuals. A similar scenario is well-established with the insulin gene (*Ins*) in T1D [120, 121]. A Variable Number of Tandem Repeats (VNTR) 5’ of *Ins *that are predisposing to T1D cause an increase in mRNA expression of *Ins* within the pancreas but a reduction in thymus tissue [120]. The reduced expression in thymus may affect the education of maturing T cells to insulin, resulting in the production of insulin auto-reactive T cells in the periphery. Further *TSHR* expression studies with reference to GD associated genotypes in thymus tissue are required to investigate the possibility of a similar role in GD. It should be noted that peptide binding studies have shown that GD associated HLA class II variants are able to bind peptides belonging to TSHR and Tg proteins [111, 112]. These studies have however, not accounted for post-translational modifications or influences by other environmental factors on peptide antigenicity, which has been observed in other autoimmune diseases, such as citrullination of proteins in RA [122]. 

The T lymphocytes (T cells) have been implicated in AITD pathophysiology for a long time, with susceptibility genes, *CTLA-4 *and *PTPN22* both expressed in various T cell subsets. CTLA-4 knock out (KO) mice exhibit fatal lymphocytic infiltration of multiple organs within the first 3-4 weeks of life, suggesting CTLA-4 is involved in the control of lymphocyte activation and proliferation [123, 124]. Previous studies suggested CTLA-4 functions *via *its APC ligands CD80 and CD86 to increase the activation threshold for self-antigens in Effector T cells [125, 126]. Although more recent studies in mice have demonstrated that CTLA-4 is also essential in regulatory T cell function (Treg) (Foxp3^+^) where CTLA-4-deficient-Tregs have diminished ability to suppress APC mediated T cell activation [127]. Furthermore, CTLA-4 has been shown to play an important role in thymic development of Tregs, where CTLA-4 deficiency causes an increased frequency of self-reactive Tregs in the periphery [128]. While CTLA-4 may have diverse functions in different cell types, recent studies have characterised at least one molecular mechanism, where CTLA-4 takes up and subsequently degrades CD80/CD86 ligands by endocytosis from the APC surface in various T cell subsets including Tregs [129]. The CD80/86 ligands are shared by the T cell activator CD28, and therefore, depletion of ligands by CTLA-4, inhibits CD28 mediated T cell activation. Another example of this is *PTPN22*, which encodes the T cell signalling molecule LYP. LYP interacts with various cell signalling molecules, like Csk-kinase, c-Cbl and Grb-2 to inhibit T cell activation [67, 130]. The R620W polymorphism is most likely the causal variant within this gene and it was assumed that the AITD associated LYP-TRP620 confers a loss of function in LYP, and therefore, a lower T cell activation threshold [68]. However, it has been demonstrated that the AITD associated LYP-TRP620 causes reduced activity of T cells and a lower expression of the T cell activation marker IL-2 following stimulation of TRP620 carrying T cells [131]. The LYP-TRP620 variant has also demonstrated reduced activity in B cells thus also highlighting its role in other cell types [132]. Another T cell marker associated with GD is *IL2Rα*; a key marker used to characterise CD4^+^CD25^+^Foxp3^+^ T regs [74]. The role of IL-2R signalling in GD pathogenesis recently gathered further support, with association of the IL-2 gene region with GD [103]. In T1D, highly associated *IL2Rα* variants are also associated with lower levels of soluble IL2Rα protein [78]. While this remains to be investigated in GD, it is likely that aberrant polymorphisms in molecules involved in IL-2R signalling may predispose to GD, possibly by affecting Treg function or other T cell subsets. The various AITD susceptibility genes known to be involved in many aspects of T cell function, highlights a prominent role for T cell mediated pathways in GD pathogenesis.

The production of thyroid specific autoantibodies by auto-reactive B cells in AITD, obviously suggests a link to B cell mediated immune mechanisms. Recently there has been a growing number of AITD susceptibility genes associated with B cell biology, including; *CD40* [87-89], *SCGB3A2* [51, 52, 65] and *FCRL3* [96-98]. Genetic variations in these genes may disrupt aspects of B cell function, such as cell signalling *via *FCRL3, leading to thyroid specific autoantibody production. While B cells were previously thought to primarily function as APCs and effector cells, there is emerging evidence of B cell involvement in adaptive immunity, particularly with the identification of regulatory B cells [133]. Indeed it has been shown that subgroups of B cells may regulate T cell activation and promote the proliferation of Tregs [134]. The improved understanding of B cell biology combined with the increasing number of AITD susceptibility loci expressed within these cells, may escalate future interest in B cells as a potential therapeutic target. 

## CONCLUSIONS

The search for AITD susceptibility loci has evolved considerably over the years with the majority of risk genes identified or confirmed within the last decade. The field of genetics is currently undergoing a renaissance in terms of our understanding of human genetic architecture and our ability to screen for disease risk with ever more cost-effective platforms. The future challenge in AITD genetics is to perform extensive surveys of genetic association across the entire spectrum of type and frequency of genetic variation. In the next few years we hope to possess a more complete picture of AITD genetic risk, which will undoubtedly bring new challenges. A more complete inventory of AITD risk genes will be vital to develop more precise disease-models of AITD pathogenesis, which in future might be translated to improve disease management. 

## Figures and Tables

**Table 1.** Shows Both Confirmed Graves’ disease Susceptibility Loci and Regions Requiring Further Confirmation of Association. Table Adapted from Simmonds and Gough (2011) [166]

Chromosome	Gene/Region	References
** Confirmed GD Susceptibility Loci**		
6p21	HLA Class II	Heward *et al*. (1998) [28], Chen *et al*. (2000) [29], Ban *et al*. (2004) [44], Simmonds *et al*. (2005) [43], Wongsurawat *et al*. (2006) [135]
6p21	HLA Class I	Farid *et al*. (1976) [136], Grumet *et al*. (1974) [137], Mather *et al*. (1980) [138], Huang *et al*. (2003) [139], Simmonds *et al*. (2007) [42]
2q33	* CTLA-4*	Yanagawa *et al*. (1995) [27], Vaidya *et al*. (1999) [140], Ueda *et al*. (2003) [9], Furugaki *et al*. (2004) [141]
1p13	* PTPN22*	Smyth *et al*. (2004) [69], Velaga *et al*. (2004) [70], Heward *et al*. (2007) [73]
10p15	* IL2Rα*	Brand *et al*. (2007) [10]
14q31	* TSHR*	Hiratani *et al*. (2005) [57], Dechairo *et al*. (2005) [56], Brand *et al*. (2009) [58]
5q32	* SCGB3A2*	Song *et al*. (2009) [51], Simmonds *et al*. (2010) [65], Chistiakov *et al*. (2011) [52]
20q13	* CD40*	Tomer *et al*. (2002) [89], Simmonds *et al*. (2005) [142], Houston *et al*. (2004) [87], Jacobson *et al*. (2005) [88], Jacobson *et al*. (2007) [143]
1q22	* FCRL3*	Kochi *et al*. (2005) [97], Simmonds *et al*. (2006) [144], Owen *et al*. (2007) [145]
8q24	* Tg*	Tomer *et al*. (2002) [86], Collins *et al*. (2003) [83], Collins *et al*. (2004) [84], Tomer *et al*. (2004) [85]
** Implicated Risk Loci Requiring Confirmation **		
2p25	* TPO*	Kotani *et al*. (1986) [146], Kotani *et al*. (1986) [147], Ludgate *et al*. (1990) [148], Pirro *et al*. (1995) [149]
2q24	* IFIH1*	Sutherland *et al*. (2007) [150], Todd *et al*. (2007) [103]
14q31	* DIO2*	Chistiakov *et al*. (2004) [151]
5q32	* ADRB2*	Chu *et al*. (2009) [64]
1q23	* FCGRIIa*	Yesmin *et al*. (2010) [152]
1p31	* IL23R*	Huber *et al*. (2008) [153]
5q31	* IL-3*	Chu *et al*. (2009) [66], Simmonds *et al*. (2010) [169]
5q31	* IRF1*	Yang *et al*. (2005) [61]
6p21	* PSMB8*	Vives-pi *et al*. (1997) [154], Heward *et al*. (1999) [155]
6p21	* PSMB9*	Vives-pi *et al*. (1997) [154], Heward *et al*. (1999) [155]
2q37	* PDCD1*	Newby *et al*. (2007) [156]
6p21	* TNFα*	Simmonds *et al*. (2004) [157], Nakkuntod *et al*. (2006) [158]
7q34	* TCR-β*	Demaine *et al*. (1989) [159], Pickerill *et al*. (1993) [160], Zhang *et al*. (2000) [161]
Xp11	* FOXP3*	Owen *et al*. (2006) [91], Ban *et al*. (2007) [90]
5q31	* IL-13*	Bednarczuk *et al*. (2003) [63], Hiromatsu *et al*. (2005) [162], Simmonds *et al*. (2005) [62]
5q31	* IL-4*	Heward *et al*. (2001) [163], Yang *et al*. (2005) [61]
2q13	* IL-1RN*	Blakemore *et al*. (1995) [164], Muhlberg *et al*. (1998) [165]
6p21	* LTA*	Nakkuntod *et al*. (2006) [158]
12q13 8q23	*VDR* *SAS-ZFAT*	Ban *et al*. (2000) [166], Ban *et al*. (2000) [167], Collins *et al*. (2004) [92], Ramos-Lopez *et al*. (2005) [93], Stefanic *et al*. (2005) [94] Shirasawa *et al*. (2004) [168]
